# Severe pneumonia caused by *Chlamydia abortus* complicated by hemophagocytic syndrome: a case report

**DOI:** 10.3389/fmed.2025.1547766

**Published:** 2025-03-11

**Authors:** Jisong Xu, Haiwen Zeng, Huangen Li, Xiaoyun Lin, Tianlai Lin

**Affiliations:** Department of Critical Care Medicine, Quanzhou First Hospital Affiliated to Fujian Medical University, Quanzhou, China

**Keywords:** *Chlamydia abortus*, pneumonia, metagenomic next-generation sequencing, hemophagocytic lymphohistiocytosis, case report

## Abstract

**Background:**

Pneumonia caused by *Chlamydia abortus* (*C. abortus*) is uncommon, particularly when complicated by severe acute respiratory distress syndrome (ARDS) and multiple organ dysfunction syndrome (MODS). Hemophagocytic lymphohistiocytosis (HLH) is a rare and potentially fatal disease characterized by the uncontrolled activation and non-malignant expansion of macrophages and T lymphocytes. This report describes a case of severe pneumonia complicated by hemophagocytic lymphohistiocytosis, caused by *Chlamydia abortus*.

**Case introduction:**

A 42-year-old female with no history of underlying medical conditions, no known exposure to poultry or avian animals, and no consumption of undercooked sheep or ewes contaminated with infected placenta, presented to the respiratory medicine department with a 3-day history of fever, cough, and sputum production. Initially diagnosed with community-acquired pneumonia, she was treated with piperacillin-tazobactam for 5 days. However, despite 12 h of high-flow oxygen therapy, her oxygenation did not improve, and she was transferred to the ICU, where she received additional treatments, including moxifloxacin and methylprednisolone. Her condition worsened further, prompting the initiation of veno-venous extracorporeal membrane oxygenation (VV-ECMO) and bronchoalveolar lavage for metagenomic next-generation sequencing (mNGS) analysis. The mNGS results identified *Chlamydia abortus* with a count of 180,791, leading to the cessation of moxifloxacin and the addition of omadacycline to her regimen. After 13 days of ECMO therapy, her condition improved, and the ECMO was discontinued. The endotracheal tube was successfully removed 15 days after intubation. However, 3 days later, the patient developed recurrent fever, pancytopenia, elevated ferritin, blood lipids, soluble CD25, and decreased natural killer cell activity, leading to a diagnosis of hemophagocytic lymphohistiocytosis (HLH). She was treated with ruxolitinib, etoposide, and other supportive medications. Despite treatment, her condition continued to deteriorate. Three days later, the family opted to discontinue therapy due to financial constraints. She passed away 12 h later.

**Conclusion:**

*Chlamydia abortus* infection can result in severe acute respiratory distress syndrome (ARDS), necessitating prompt diagnosis and active clinical intervention. This case is unique due to the rare occurrence of HLH following *Chlamydia abortus* infection, a pathogen not commonly associated with this condition. Metagenomic next-generation sequencing (mNGS) offers a distinct advantage in rapidly and accurately identifying rare pathogen infections, while extracorporeal membrane oxygenation (ECMO) can be an effective treatment for severe pneumonia caused by *Chlamydia abortus*. It highlights the importance of early recognition and management of HLH in patients with severe, unexplained infections, particularly in those with unusual pathogens. Additionally, *Chlamydia abortus* infection may be complicated by HLH. Clinicians should remain vigilant for patients presenting with unexplained high fever, hepatosplenomegaly, and pancytopenia, and HLH screening should be initiated promptly. Early intervention can significantly improve patient survival rates.

## Introduction

*Chlamydia abortus* is a zoonotic pathogen known to infect a wide range of animals, such as goats, sheep, yaks, pigs, horses, rabbits, guinea pigs, mice, and various farmed fur-bearing species (1–4). Additionally, *Chlamydia abortus* is responsible for causing abortion, stillbirth, gestational sepsis, and pelvic inflammatory disease in humans (5–8). In recent years, there have been increasing reports of pneumonia caused by *Chlamydia abortus* (9–12). Of these, only one case was treated with extracorporeal membrane oxygenation (ECMO) (9).

Hemophagocytic lymphohistiocytosis (HLH) is a potentially life-threatening condition marked by severe inflammation, which is driven by the excessive release of inflammation cytokines from hyperactive lymphocytes and macrophages. The disorder presents with a wide range of clinical manifestations, including fever, hepatosplenomegaly, pancytopenias, hypertriglyceridemia, hypofibrinogenemia, neurological symptoms, and histopathological evidence of hemophagocytosis in the bone marrow or other affected organs (13). Infection caused by various pathogens, including viruses, bacteria, and parasites, have been implicated in the development of HLH, leading to the classification of the condition as infection-associated HLH (14–16). At present, there have been no reports of abortus chlamydia infection complicated with HLH.

Here, we describe a case of severe pneumonia caused by *Chlamydia abortus* with hemophagocytic lymphocytosis that presented with refractory ARDS and was treated with ECMO. In addition, moxifloxacin and omadacycline were used to treat *Chlamydia abortus* infection, and a combination of ruxolitinib and etoposide was used to treat HLH.

## Case presentation

**Figure 1:**
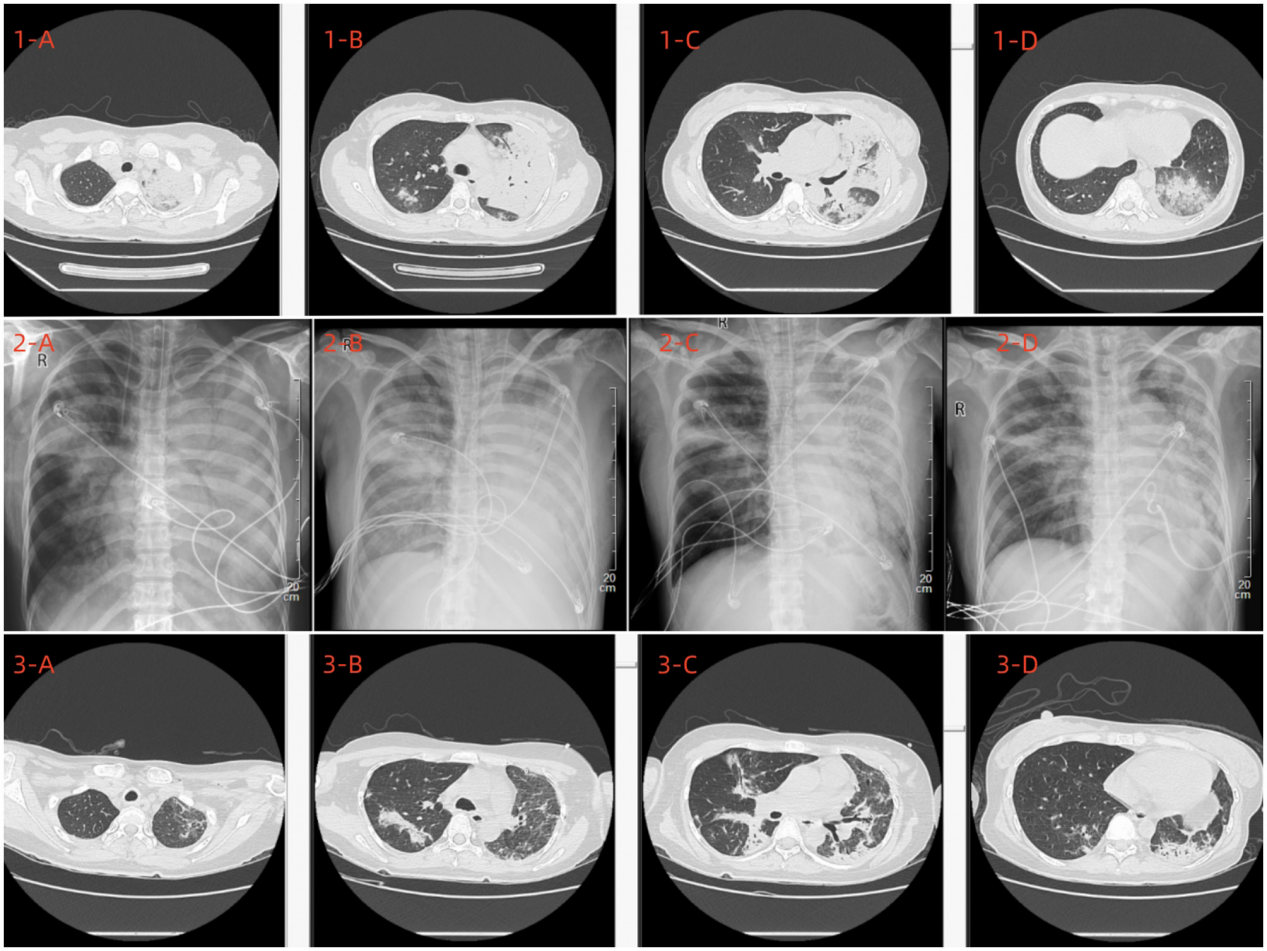
Upon admission, a chest CT scan **(1A–D)** revealed multiple exudative lesions throughout both lungs, predominantly in the left lung. **(2A–D)** Depict the chest X-ray findings on the 6th, 8th, 15th, and 18th days post-admission, respectively. A subsequent chest CT conducted on the 22nd day post-admission **(3A–D)** indicated a significant reduction in the size of the lesions in both lungs.

A 42-year-old female bank clerk with no history of smoking and no reported exposure to poultry or avian animals presented to the Department of Respiratory Medicine at our hospital with a 3-day history of fever, cough, and sputum production. The patient never had consumed uncooked ewe or sheep meat contaminated with infected placenta. On admission, physical examination revealed a body temperature of 40°C, a pulse rate of 112 beats per minute, a respiratory rate of 20 breaths per minute, and a blood pressure of 115/59 mmHg. Auscultation detected scattered bilateral fine crackles. Laboratory findings included a white blood cell count of 12.01 × 10^9^/L, neutrophil count of 10.32 × 10^9^/L, lymphocyte count of 1.13 × 10^9^/L, hemoglobin level of 104 g/L, platelet count of 217 × 10^9^/L, erythrocyte sedimentation rate of 58 mm/h, C-reactive protein level of 87.56 mg/L, and procalcitonin level of 0.219 ng/mL, Liver and renal function were normal, and electrolytes showed K 4.0 mmol/L, Na 138 mmol/L, Cl 102 mmol/L. LDH was 260 U/L. Chest CT imaging demonstrated multiple patchy hyperdense opacities and consolidation in both lungs (Figure 1, 1A–D). Sputum, blood, and urine cultures were negative for pathogenic bacteria. Viral panel testing for influenza A and B viruses, adenovirus, respiratory syncytial virus, parainfluenza virus, metapneumovirus, COVID-19, Mycoplasma pneumoniae, and Chlamydia were all negative. Following admission, the patient was administered oxygen via nasal cannula at 3 L/min, piperacillin-tazobactam (4.5 g intravenously every 8 h), and methylprednisolone (40 mg intravenously once daily).

After 5 days of treatment, the patient's condition failed to improve, and her respiratory status deteriorated. Despite 12 h of high-flow nasal cannula (HFNC) ventilation (oxygen concentration: 80%, flow rate: 50 L/min), her respiratory function remained compromised, with oxygen saturation levels fluctuating between 70 and 85%. Subsequent arterial blood gas analysis revealed a pH of 7.54, a partial pressure of oxygen (PaO2) of 46 mmHg, a partial pressure of carbon dioxide (PaCO2) of 30 mmHg, and a lactate level of 3.6 mmol/L. These findings indicated the presence of severe acute respiratory distress syndrome (ARDS). Consequently, endotracheal intubation and mechanical ventilation were urgently initiated, and she was promptly transferred to the intensive care unit (ICU) of our hospital. The initial mechanical ventilation parameters are as follows: VC-AC mode, FiO2: 100%, respiratory rate: 14 breaths/min, tidal volume: 400 mL, PEEP: 12 cmH2O. Concurrently, we have implemented prone ventilation, deep analgesic sedation, and lung-protective ventilation strategies.

**Figure 2:**
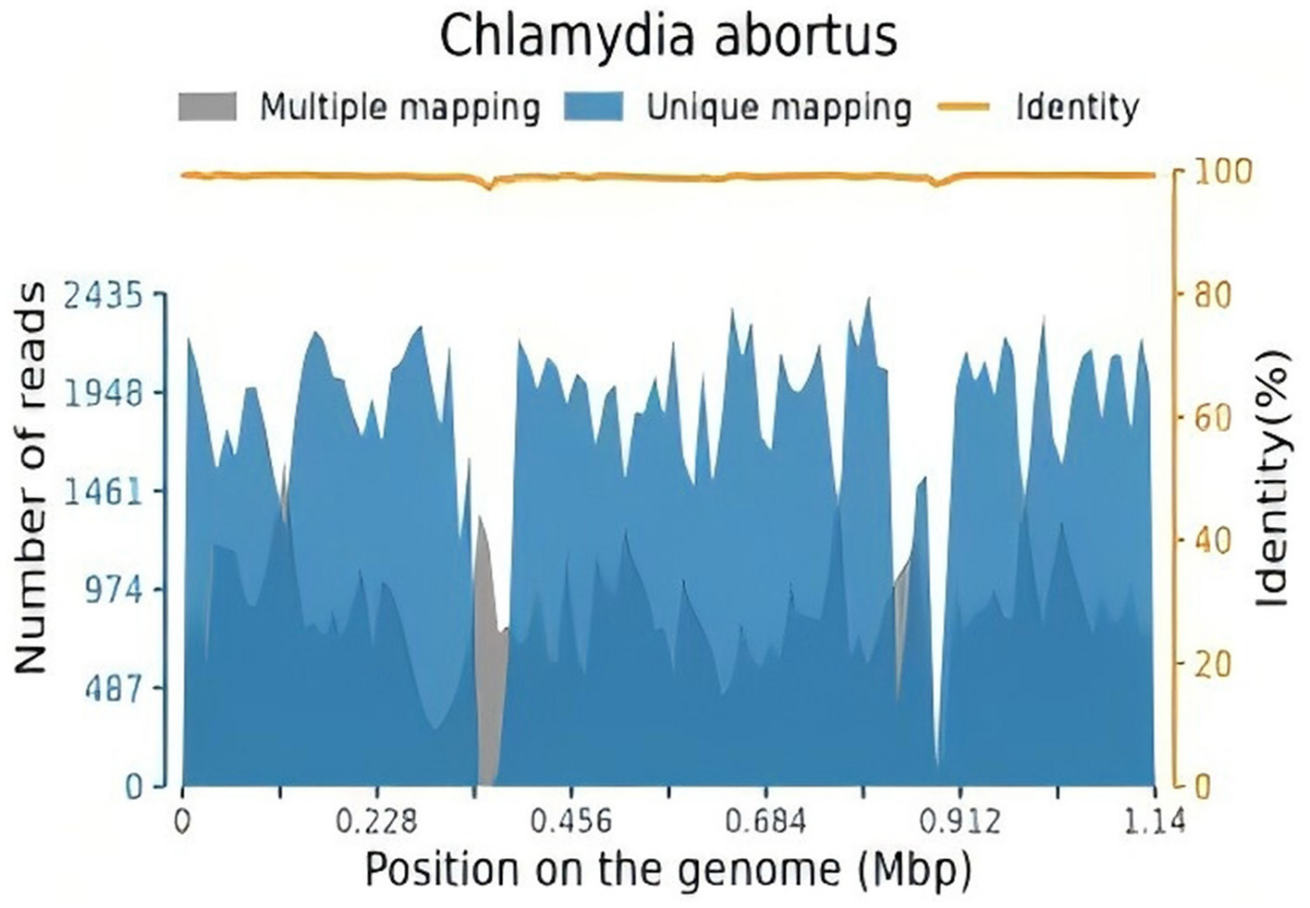
The total number of base pairs in the genome of this species is 1,143,694 (bp), the total length of the sequence of this species covered is 1,051,398 (bp), with a coverage of 91.93%, and an average depth of 11.561 X.

**Figure 3:**
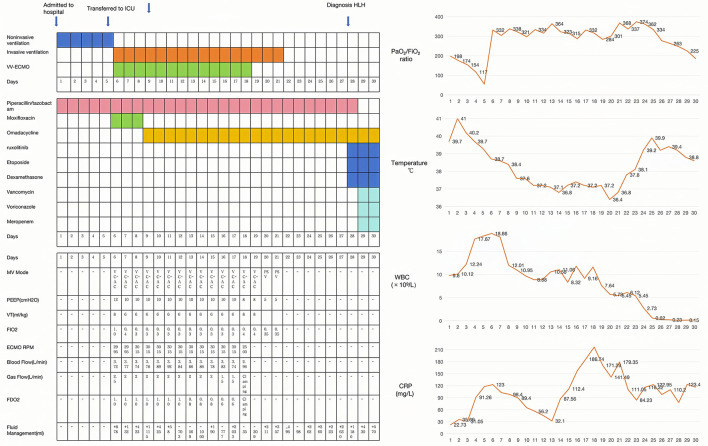
Hospitalization timeline and clinical treatments.

After 10 h of mechanical ventilation, Arterial Blood Gas (ABG) revealed a pH of 7.30, a Partial Pressure of Oxygen (PaO2) of 55 mmHg, and a Partial Pressure of Carbon Dioxide (PaCO2) of 47 mmHg. Given the diagnosis of acute respiratory distress syndrome (ARDS) with a PaO2/FiO2 ratio < 80 mmHg persisting for more than 6 h, a veno-venous extracorporeal membrane oxygenation (VV-ECMO) system was initiated. The initial settings for VV-ECMO were a pump speed of 3,000 RPM, a blood flow rate of 4.2 L/min, and an airflow rate of 2 L/min. Vascular access was established via the right femoral vein. Ventilatory parameters were adjusted to a tidal volume of 6 mL/kg, a positive end-expiratory pressure (PEEP) of 10 cmH2O, and a respiratory rate of 14 breaths per minute. To identify the underlying etiology, bronchoalveolar lavage fluid (BALF) was collected for metagenomic next-generation sequencing (NGS) analysis. The initial antimicrobial regimen included piperacillin-tazobactam (4.5 g intravenously every 8 h) and moxifloxacin (0.4 g intravenously every 8 h). Two days later, mNGS of the BALF identified Chlamydia abortus with a sequence count of 180,791 and a relative abundance of 93.399% (Figure 2). Considering the potential for co-infection, omadacycline (0.1 g intravenously daily) was added to the treatment regimen, while piperacillin-tazobactam (4.5 g intravenously every 8 h) was continued. Concurrently, VV-ECMO, mechanical ventilation, analgesic sedation, and prone positioning were maintained. The patient's oxygenation progressively improved, and follow-up chest X-rays demonstrated gradual resolution of pulmonary exudates (Figure 1, 2A–D). The ECMO was successfully weaned after 326 h, and endotracheal intubation was discontinued 2 days later. High-flow oxygen therapy was subsequently initiated. A follow-up chest CT scan revealed significant resolution of lung lesions (Figure 1, 3A–D). The patient's clinical course is summarized in Figure 3.

Despite the improvement in the patient's lung condition, 3 days after the removal of endotracheal intubation, the patient experienced a recurrence of high fever and developed pancytopenia. Laboratory findings revealed a white blood cell count of 0.12 × 10^9^/L, neutrophil count of 0 × 10^9^/L, lymphocyte count of 0.12 × 10^9^/L, hemoglobin level of 81 g/L, and platelet count of 71 × 10^9^/L. A bone marrow aspirate was subsequently performed, which demonstrated hemophagocytosis. Given these findings, a diagnosis of hemophagocytic lymphohistiocytosis (HLH) was suspected. Further laboratory investigations confirmed elevated levels of ferritin (>15,000 ng/mL), fibrinogen (1.8 g/L), triglycerides (3.12 mmol/L), lactate dehydrogenase (5,143 U/L), interleukin-6 (250 pg/mL), and soluble interleukin-2 receptor (sCD25; 4,790 U/mL; reference range: 223–710 U/mL). Additionally, natural killer (NK) cell activity was reduced to 13.23% (reference range: 15.11–23.7%). On day 4 after the detection of pancytopenia (day 28 of admission), following a series of laboratory tests, the patient was diagnosed with hemophagocytic lymphohistiocytosis (HLH). The patient was treated with a combination of ruxolitinib (100 mg twice daily) and the HLH-1994 protocol, which included etoposide (100 mg on days 2 and 15) and dexamethasone (10 mg once daily). The patient developed neutropenia and presented with persistent high fever, significantly elevated inflammatory markers such as CRP, and the need to consider the possibility of concurrent severe infections caused by other pathogens, including bacteria and fungi. According to the Infectious Diseases Society of America (IDSA) guidelines, it is recommended to initiate empirical broad-spectrum antibiotic therapy for patients with neutropenia and fever. Therefore, after obtaining appropriate microbiological samples, we adjusted the antimicrobial treatment plan accordingly. The antimicrobial regimen was adjusted to omadacycline (0.1 g intravenous infusion every 12 h), vancomycin (1 g intravenous infusion every 12 h), meropenem (1 g intravenous infusion every 8 h), and voriconazole (200 mg intravenous infusion every 12 h). Despite these interventions, the patient continued to experience recurrent fever and failed to exhibit an increase in white blood cell count. Unfortunately, 3 days into the chemotherapy regimen, the patient's family elected to discontinue further treatment due to financial constraints. The patient passed away 12 h after discharge.

## Discussion

*Chlamydia abortus* is the most significant chlamydial affecting animal husbandray. This obligate intracellular Gram-negative bacterium infects a variety of animal species, with cattle, sheep, pigs, and several poultry types serving as its natural hosts. In these animals, it is predominantly linked to the occurrence of miscarriages (1–4). Additionally, *Chlamydia abortus* can be transmitted via the respiratory route and has the potential to infect humans through direct contact, leading to atypical pneumonia, miscarriage in pregnant women, and stillbirths. Consequently, it is classified as a zoonotic pathogen (5–7). Individuals such as farmers, veterinarians, and those frequently exposed to cattle, sheep, and poultry are at a higher risk of contracting *Chlamydia abortus*-induced pneumonia. In the present case, the patient lacks a history of exposure to such animals, and none of her family members exhibit similar symptoms.

Currently, pulmonary infections caused by *Chlamydia abortus* are rare. Through a literature review, we identified nine cases of pneumonia attributable to *Chlamydia abortus* from China (9–12), among which six cases resulted in respiratory failure, five cases exhibited liver damage, eight cases had favorable outcomes, and one case discontinued treatment due to financial constraints. Consequently, early diagnosis and timely intervention are critical.

At present, no serological tests have been approved for the specific diagnosis of *Chlamydia abortus* infection in humans. As a result, Polymerase Chain Reaction (PCR) remains the primary method for diagnosing this infection (17). Nevertheless, this molecular method is particular and relies on certain prior assumptions. It is generally employed only when clinicians suspect an infection with *Chlamydia abortus* (9). In this case, traditional methods did not identify any pathogenic bacteria in the sputum, which complicated accurate diagnosis and treatment. Next-generation sequencing (NGS), however, is an unbiased technique capable of detecting all potential pathogens in clinical samples. This makes it particularly valuable for identifying novel, rare, and atypical causes of complex infectious diseases (18). Moreover, NGS is known for its rapid detection capabilities, and its results are less influenced by previous antibiotic treatments (19). Therefore, we performed NGS and finally clarified that the pathogen was *Chlamydia abortus* in our patient. *Chlamydia abortus* infection, though rare, can be severe and life-threatening, as demonstrated in our case where the patient developed MODS and experienced rapid deterioration. Thus, prompt and accurate treatment is critical. Chlamydia species are typically susceptible to antibiotics that inhibit DNA and protein synthesis, such as tetracyclines, macrolides and quinolones (20). To date, no standardized treatment guidelines have been established for *Chlamydia abortus* infections in humans. Recent *in vitro* studies have indicated that doxycycline may offer therapeutic benefits in eliminating *Chlamydia abortus* infection in epithelial cells (21). Moreover, macrolide antibiotics are regarded as the preferred alternative for patients who cannot use tetracyclines. *In vitro* studies have also demonstrated the efficacy of macrolides, such as azithromycin, against *Chlamydia psittaci* (22, 23). Fang et al. reported that omadacycline, a novel tetracycline, could offer a promising treatment option for severe *Chlamydia psittaci* infections, owing to its favorable safety profile and the absence of the need for dose adjustments in special populations (24). In some instances, quinolones can be effective; however, their efficacy is generally lower than that of tetracyclines and macrolides (23). The patient presented in this study had initially been treated with piperacillin/tazobactam and moxifloxacin, but his pulmonary function continued to deteriorate, indicating poor efficacy or insufficient treatment. Given the therapeutic options described in the literature, the patient's condition, and the possibility of mixed infection caused by chlamydia, we opted for a combination therapy of omadacycline and piperacillin/tazobactam. This patient's condition improved clinically and biologically. ECMO was successfully discontinued, and the endotracheal tube was subsequently extubated. However, she was concurrently diagnosed with HLH.

HLH is a rare yet potentially fatal disorder of immune regulation, initially identified in 1939 by Scott and Robb-Smith, who referred to it as histiocytic medullary reticulosis (25). The disease is characterized by (1) excessive activation of CD8+ T lymphocytes and macrophages; (2) the abnormal proliferation, migration, and infiltration of these cells into multiple organs; and (3) hypercytokinemia, with elevated cytokine levels leading to progressive organ dysfunction (26). Clinically, HLH is primarily characterized by persistent fever, hepatosplenomegaly, disseminated intravascular coagulation, and cytopenia (involving at least two cell lineages). It can be classified based on the underlying cause into genetic or acquired types. The genetic form is linked to various genetic abnormalities and typically manifests in infancy or childhood. In contrast, acquired HLH is more common in adults, although both forms can affect individuals across all age groups. Acquired HLH is a highly heterogeneous condition that can be triggered by a range of factors, including infections, malignancies, and metabolic or autoimmune diseases (26–28). Diagnosing HLH during the acute phase is both complex and challenging for clinicians due to the nonspecific nature of its presentation and the absence of a gold standard for diagnostic criteria. In 1991, the Familial Hemophagocytic Lymphohistiocytosis (FHL) study group of the Histiocyte Society established diagnostic guidelines, which were later revised in the HLH-2004 trial (10). The patient currently fulfills seven out of the eight diagnostic criteria for HLH as outlined in the HLH-2004 protocol, including fever, cytopenias, elevated ferritin levels, hypertriglyceridemia, hemophagocytosis observed in the bone marrow, reduced natural killer (NK) cell activity, and increased soluble CD25 (sCD25) levels. The HLH-2004 diagnostic criteria were initially developed for research purposes. Although studies on external validity demonstrate a sensitivity and specificity of around 90%, these criteria still have certain limitations (11). A notable limitation is the inability of many centers to conduct advanced tests, such as measuring soluble CD25 levels, which often require outsourcing to external laboratories, leading to a delay of at least 3–4 days. Recent data suggest that modifying the diagnostic criteria for HLH in adults could offer a more flexible and comprehensive approach (29).

Various infectious agents, including viruses, bacteria, fungi, and parasites, have been linked to the development of HLH (14–16). Viral infections are the most commonly reported cause of HLH, with Epstein-Barr virus (EBV) being considered the most frequent trigger. EBV-associated HLH typically presents as an acute onset and is closely related to an excessive immune response (30). In contrast, HLH triggered by bacterial infections is relatively rare; however, there have been reports of Mycoplasma pneumoniae infection leading to HLH, particularly in pediatric patients (31, 32). *Chlamydia abortus*, as an intracellular pathogen, can trigger a strong inflammatory response by activating macrophages and T lymphocytes, which may be one of the key mechanisms underlying the development of HLH. In this case, despite the control of pulmonary infection following ECMO treatment, the patient developed typical symptoms of HLH, including persistent fever, pancytopenia, hyperferritinemia, and hemophagocytosis in the bone marrow. This suggests that *Chlamydia abortus* infection may induce HLH by overactivating the immune system, leading to a cytokine storm. Although no cases have been reported in the literature linking *Chlamydia abortus* infection directly to HLH, this case highlights the need for clinicians to be alert to the possibility of HLH in patients with severe *Chlamydia abortus* infections, particularly when unexplained fever, pancytopenia, and hepatosplenomegaly are present. Timely HLH screening should be conducted in such cases.

First-line treatment strategies for HLH predominantly follow the HLH-94 and HLH-2004 protocols, which recommend a combination of immunosuppressive agents including dexamethasone, etoposide, and cyclosporine A (33–35). In this particular case, the patient was administered ruxolitinib in conjunction with the HLH-1994 regimen post-diagnosis; however, the treatment was ineffective due to the rapid progression of the disease. Given that HLH is a highly aggressive and fatal condition, the timely initiation of appropriate therapeutic interventions is crucial for improving outcomes (35). Nevertheless, the management of infection-associated HLH is more intricate, necessitating the simultaneous control of the inflammatory response and the effective eradication of pathogens, thereby demanding a more comprehensive and rigorous approach to treatment.

The tragic outcome of this case underscores the devastating nature of hemophagocytic lymphohistiocytosis (HLH), particularly when compounded by severe infection, as it carries an extremely poor prognosis. The outlook for HLH patients is closely tied to early disease recognition, timely and effective therapeutic measures, and the patient's underlying health condition. Clinicians encounter substantial challenges in managing HLH secondary to *Chlamydia abortus* infection due to its rarity and the complexity of its clinical manifestations. In the context of a severe infectious etiology, patients presenting with unexplained high fever, hepatosplenomegaly, and pancytopenia should raise a strong suspicion for HLH, warranting immediate investigation and intervention. Prompt diagnosis of HLH coupled with timely and appropriate therapeutic strategies can significantly enhance patient outcomes.

## Data Availability

The original contributions presented in the study are included in the article/supplementary material, further inquiries can be directed to the corresponding author.
